# The association between vaccine hesitancy and pertussis: a systematic review and meta-analysis

**DOI:** 10.1186/s13052-023-01495-8

**Published:** 2023-07-13

**Authors:** Yuning Wang, Naiyang Shi, Qiang Wang, Liuqing Yang, Tingting Cui, Hui Jin

**Affiliations:** 1 Department of Epidemiology and Health Statistics, School of Public Health, Southeast University, Nanjing, 210009 China; 2 Key Laboratory of Environmental Medicine Engineering, Ministry of Education, School of Public Health, Southeast University, Nanjing, 210009 China

**Keywords:** Vaccine hesitancy, Pertussis, Vaccine effectiveness, Children

## Abstract

**Background:**

Robust routine immunization schedules for pertussis-containing vaccines have been applied for years, but pertussis outbreaks remain a worldwide problem. This study aimed to investigate the association between vaccine hesitancy and pertussis in infants and children.

**Methods:**

We searched PubMed, Cochrane, Web of Science, Embase, and China National Knowledge Internet for studies published between January 2012 and June 2022. This study included case–control and cohort studies that assessed the association between childhood/maternal vaccine hesitancy and odds ratios (ORs), risk ratios (RRs), and vaccine effectiveness (VE) related to pertussis in infants and children $\le$ 9 years old. ORs/VEs with a 95% confidence interval (CI) were calculated. Random-effects meta-analysis models were used for appropriate pooled estimates, and heterogeneity was assessed using ${I}^{2}$. Cumulative meta-analysis and subgroup analyses stratified by study characteristics were performed.

**Results:**

Twenty-two studies were included, with a mean quality score of 7.0 (range 6.0–9.0). Infants and children with pertussis were associated with higher vaccine hesitancy to all doses (OR = 4.12 [95% CI: 3.09–5.50]). The highest OR was between children who were unvaccinated over four doses and children who were fully vaccinated (OR = 14.26 [95%CI: 7.62–26.70]); childhood vaccine delay was not statistically significantly associated with pertussis risk (OR = 1.18 [95% CI: 0.74–1.89]). Maternal vaccine hesitancy was associated with significantly higher pertussis risk in infants aged 2 and 3 months old, with higher pertussis ORs in infants $\le$ 2 months old (OR = 6.02 [95%CI: 4.31–8.50], OR = 5.14 [95%CI: 1.95–13.52] for infants $\le$ 2 and $\le$ 3 months old, respectively). Maternal and childhood VEs were high in reducing pertussis infection in infants and children. The administration time of maternal vaccination had little effect on VE.

**Conclusion:**

Vaccine hesitancy increased pertussis risks in infants and children. Ensuring that children receive up-to-date pertussis vaccines is essential; short delays in receiving childhood vaccinations may be unimportant. Maternal vaccinations for pertussis should be encouraged.

**Supplementary Information:**

The online version contains supplementary material available at 10.1186/s13052-023-01495-8.

## Introduction

Routine childhood vaccination with vaccines against pertussis has been a long-standing immunization program in several countries [1]. Since the resurgence of pertussis in the early 2010s [2–4], several countries have recommended Tdap/dTap for women between 28 and 38 weeks of pregnancy [5–9]. However, pertussis outbreaks continue to be reported worldwide [10–13], with high increments in incidence among fully vaccinated children [14–16]. This may be due to a lack of antibody protection in new-born infants [17], or the waning immunity of DTaP over time [18]. Thus, strategic vaccine schedules for children and timely uptake of boosters are important [19]. Studies estimating vaccination coverage without investigating the timeliness may mask the delay of vaccination [20, 21]. The association between vaccine hesitancy – an important factor related to delayed or missed immunizations – and pertussis in infants and children still lacks systematic investigations [22, 23].

Vaccine hesitancy is a psychological state, vaccination behavior, or decision-making process [22]. Maternal vaccine hesitancy was associated with lower pertussis-containing vaccine uptake (0–74%) [24], resulting in fewer newborns receiving maternal antibodies [25]. Parental hesitancy and non-medical exemptions have contributed to childhood vaccine hesitancy [15, 26–28]. In Northern California, the hazard ratios of pertussis were 13 and 1.9 times higher among the unvaccinated and under-vaccinated children than the fully vaccinated, respectively [19]. Children in exemption clusters were 2.5 times more likely to develop pertussis than non-exemption clusters [28]. The COVID-19 pandemic disrupted routine immunization schedules through the reduced availability of vaccine services during lockdowns, emerging confusing messages about vaccinations, and increasing reluctance to receive vaccinations [24, 29–32], leading to decreased childhood and prenatal pertussis vaccine coverage [33, 34]. Together, higher pertussis risks in infants and children may exist in the post-pandemic period.

Previous meta-analyses and systematic reviews focused only on 1) the effect of childhood unvaccination/under-vaccination on pertussis in specific countries [18], 2) the effectiveness of maternal vaccinations [35], or 3) the effectiveness of childhood pertussis-containing vaccinations [36]. Therefore, we performed a systematic review and meta-analysis assessing the association between maternal and childhood vaccine hesitancy and pertussis at the population level, to investigate the importance of on-time childhood and maternal vaccination worldwide.

## Methods

This study was performed according to Preferred Reporting Items for Systematic Reviews and Meta-Analysis (PRISMA) protocols.

### Inclusion and exclusion criteria

We used vaccination behavior, a delay in the acceptance or refusal of vaccines despite the availability of vaccination services, to represent vaccine hesitancy and stratified it into three categories: unvaccinated, under-vaccinated, and vaccine delayed group [37]. Children or pregnant women were categorized to be fully vaccinated if they receive each dose within 4 days before the minimum age at administration till 30 days after the recommended age [38], or receive the vaccine during pregnancy, respectively. Children with fewer doses than recommended for their age were regarded as under-vaccinated [19, 23, 38]. Children and pregnant women who received no vaccinations were defined as unvaccinated. In the absence of a standard definition for vaccine delay [23, 39, 40], we defined children who received the dose after 30 days of recommended age but before the recommended age for the next dose as vaccine delayed.

The inclusion and exclusion criteria were developed using the PICOS framework (Table 1). We included primary studies published between 2012 and 2022 in English or Chinese that investigated the association between vaccine hesitancy and pertussis odds ratios (ORs), relative risks (RRs), or vaccine effectiveness (VE). Infants, children $\le$ 9 years old, and pregnant women were included, and all pertussis-containing vaccines were measured. We excluded studies that are non-original, lack vaccine hesitancy status, or contain inappropriate population groups, vaccine types, and outcomes. We constrained the starting time period to 2012 to measure the effects of maternal vaccine hesitancy and check the latest pertussis trends [5, 6].

**Table 1:** Inclusion and exclusion criteria, using the PICOS framework

	**Inclusion criteria**	**Exclusion criteria**
Population	Infants and children $\le$ 9 years old; pregnant women worldwide	Children > 9 years old, teenagers, and adults; nonpregnant women
Intervention	Pertussis-containing vaccines, including aP, DTaP, Tdap, dTaP, DwPT, and DTPw	Other vaccines without pertussis-containing
Control	Fully-vaccinated children who receive the recommended doses on time; pregnant women receive pertussis-containing vaccines during pregnancy	Fully-vaccinated children but did not receive on time; pregnant women received postpartum vaccination or vaccinated before pregnancy
Outcome	Primary outcome: Odds ratio (OR) and relative risk (RR) between the fully vaccinated and vaccine hesitancy groups Secondary outcome: Vaccination effectiveness (VE)	Incidence rate ratio (IRR) and hazard ratio (HR) between the fully vaccinated and vaccine hesitancy groups
Study design	Case–control, cohort, or cross-sectional studies at the population level; case reports about pertussis outbreaks	Non-original studies (eg, reviews, meta-analysis and systematic reviews, guidelines, editorials, commentaries); randomized or non-randomized clinical trials
Other	Published in English or Chinese	Without vaccine hesitancy status; limited to individual laboratory vaccine effectiveness

### Search strategy

We searched PubMed, Cochrane, Web of Science, Embase, and China National Knowledge Internet for primary studies with terms: ([vaccine delay] OR [undervaccinat*] OR [vaccine refusal] OR [vaccine effectiveness] OR [vaccine hesitan*]) AND ([pertussis] OR [pertussis outbreak]). Records were imported into EndNote (version X9.3.3) and duplicates were removed for abstract and full-text screening. Titles, abstracts, and full-text screening were independently performed by YNW and NYS.

### Quality assessment

The quality of studies was assessed using the Newcastle–Ottawa Scale (NOS) to evaluate the risk of bias [41]. Two reviewers independently assessed each study (YNW and NYS) and conflicts were resolved by a third author. The scoring system summarized each study from three aspects: selection, comparability, and exposure. Studies were divided into three qualities based on their scores as follows: $\le$ 3, low quality; 4–6, median quality; $\ge$ 7, high quality. Data were not excluded based on study quality, but the quality informed the discussion.

### Data extraction and analysis

Two reviewers (WYN and NYS) independently extracted the data, where ORs and VEs were directly extracted and RRs were transformed into ORs before extraction [42]. Our primary outcome of interest was pertussis OR between vaccine-hesitant and fully vaccinated groups. Estimates were calculated at the 95% confidence interval (CI). Heterogeneity between studies was analyzed using ${I}^{2}$ statistics, where ${I}^{2}$ < 50% was considered statistically insignificant. Due to the high heterogeneity, DerSimonian-Laird and Sidik-Jonkman random effects were used. To explore the heterogeneity sources, we conducted subgroup analyses stratified by study region, vaccine population, vaccine hesitancy status, study population age, and number of vaccine doses. A cumulative meta-analysis was performed to investigate the temporal trends [43]. Sensitivity analysis was conducted by leave-one-out analyses to ascertain that the estimates were not driven by one of the studies.

All meta-analyses were performed and all forest plots were generated using R software (version 4.1.3).

## Results

### Search results and study characteristics

**Fig. 1:**
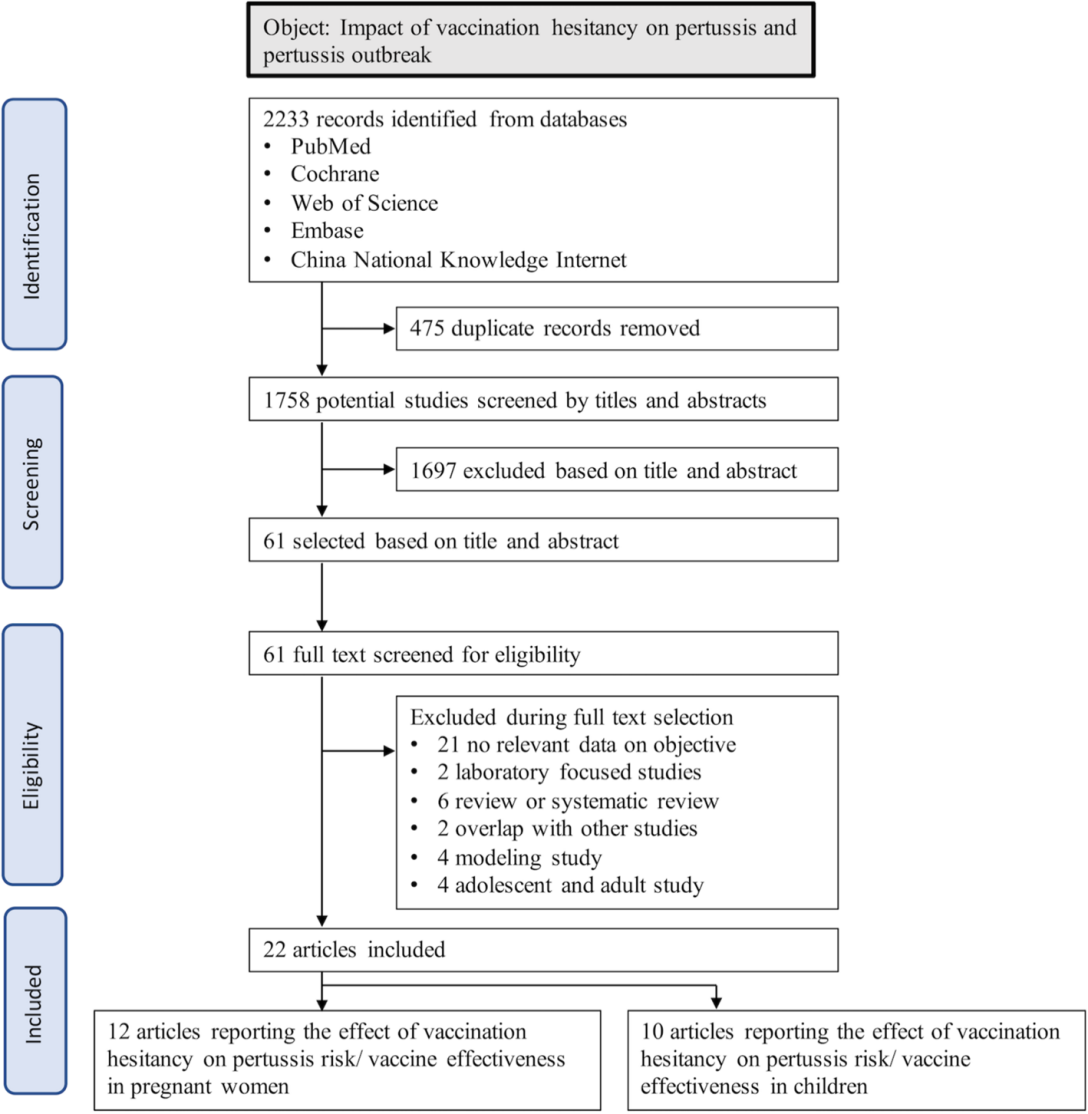
The selection flowchart of the association between vaccine hesitancy and pertussis study

A total of 2233 publications were identified, of which 475 were excluded due to duplication. 1758 titles and abstracts were screened and 1697 were excluded based on the criteria. Of the remaining 61 studies, 22 were included in the meta-analysis (cohort study = 8; case–control study = 14): 12 assessed the association between maternal vaccine hesitancy and pertussis in infants; 10 evaluated the effects of childhood vaccine hesitancy (Fig. 1). The studies were quality-appraised with a mean score of 7.0 (range 6.0–9.0), with detailed characteristics in the supplementary tables (Table S1, S2, Additional File).

Ten childhood vaccine hesitancy studies were conducted in the United States [23, 44, 45], Japan [46, 47], New Zealand [48], Canada [49], Israel [50], Peru [51], and Taiwan [40], covering Asia, North America, and South America. Six, five, and two studies measured the effects of unvaccination, under-vaccination, and vaccine delay, respectively. Twelve maternal vaccine hesitancy studies were conducted in the United States [52–55], England [56, 57], Spain [7, 58], Australia [9, 59], Brazil [60], and Argentina [8], covering Australia, Europe, and North and South America, of which ten measured the pertussis risks in infants. VEs against pertussis were investigated in all twelve studies. All included studies investigated the effects of different vaccine doses and defined pertussis according to the World Health Organization (WHO) clinical pertussis case definitions. However, various pertussis PCR laboratory confirmation methods were used across different studies – for example, Bellido-Blasco [7] included pertussis cases that lacked PCR laboratory confirmations.

### The combined effects of vaccine hesitancy

Meta-analysis of 18 studies [7–9, 23, 40, 44, 46, 47, 49, 50, 52, 53, 56–60] generated a random-effects pooled OR of 4.12 (95% CI, 3.09–5.50; *p* < 0.01; Table 2) in infants and children between the all doses vaccine-hesitant and fully vaccinated group. The cumulative meta-analysis showed a clear decreasing temporal change of pertussis OR under the effect of vaccine hesitancy (Fig. S1a,Additional file). Stratified by study region, the highest pertussis OR was in Europe and the lowest was in Eastern Asia (Table 2). Heterogeneity was insignificant in all regions except North America (${I}^{2}=$ 53%). Sensitive analysis showed that the pooled estimates were robust (Table S3, Additional File).

**Table 2:** Pertussis OR of vaccine hesitancy among different subgroups

Vaccine Population	Variable	No. of studies	Estimated OR and 95% CI	${I}^{2}$
All	**Vaccine doses**			
	all	18 [7–9, 23, 40, 44, 46, 47, 49, 50, 52, 53, 56–60]	4.12 (3.09, 5.50)	69%
	**Study Region**			
	North America	5 [23, 44, 49, 52, 53]	2.34 (1.88, 4.30)	53%
	South America	2 [8, 60]	5.18 (2.81, 9.55)	0%
	Europe	4 [7, 56–58]	10.55 (7.30, 15.26)	0%
	Australia	2 [9, 59]	3.93 (1.82, 8,49)	0%
	Eastern Asia	2 [40, 46, 47]	2.22 (1.68, 2.94)	0%
	Western Asia	1 [50]	4.58 (3.21, 6.53)	NA
Children	**Vaccine hesitancy status**			
	**Vaccine hesitant**			
	**Vaccine doses**			
	All	7 [23, 40, 44, 46, 47, 49, 50]	2.85 (2.01, 4.03)	65%
	**Study Region**			
	North America	3 [23, 44, 49]	2.60 (1.19, 5.65)	77%
	Eastern Asia	3 [40, 46, 47]	2.22 (1.68, 2.94)	43%
	Western Asia	1 [50]	4.58 (3.21, 6.53)	NA
	**Unvaccinated**			
	**Vaccine doses**			
	1	2 [45, 48]	2.04 (0.76, 5.49)	87%
	2	2 [45, 48]	3.79 (2.66, 5.40)	0%
	3	2 [45, 48]	7.79 (6.82, 8.90)	6%
	4	2 [45, 48]	14.26 (7.62, 26.70)	87%
	All	4 [46, 47, 49, 50]	4.04 (3.10, 5.27)	0%
	**Under-vaccinated**			
	**Vaccine doses**			
	1	4 [23, 40, 44, 45]	2.45 (1.69, 3.54)	29%
	2	3 [40, 44, 45]	2.45 (1.52, 3.94)	0%
	3	3 [40, 44, 45]	4.78 (2.19, 10.44)	51%
	4	2 [40, 44]	8.21 (1.26, 53.49)	61%
	All	2 [40, 44]	2.95 (1.61, 5.40)	63%
	**Number of vaccine dose**			
	Dose 1&2&3	2 [23, 40]	3.44 (1.88, 6.29)	78%
	Dose 4&5	1 [51]	4.34 (2.17–8.68)	NA^a^
	Dose 4	2 [23, 40]	2.09 (0.74, 5.90)	72%
	Dose 5	1 [23]	4.60 (2.59–8.17)	NA
	**Vaccine delayed**			
	**Number of vaccine dose**			
	All	2 [23, 40]	1.40 (0.62, 3.16)	59%
	Dose 4	1 [23]	0.80 (0.48, 1.34)	NA
	Dose 5	1 [23]	1.30 (0.48, 3.49)	NA
Pregnant Women	**Population age**			
	All	10 [7–9, 52, 53, 56–60]	5.63 (3.87, 8.18)	53%
	$\le$ 2 months	8 [8, 52, 53, 56–60]	6.05 (4.31, 8.50)	12%
	$\le$ 3 months	4 [7, 9, 53, 57]	5.14 (1.95, 13.52)	80%

^a^*NA* Not applicable

### Childhood vaccine hesitancy and pertussis

Meta-analysis generated a random-effects pooled OR of 2.85 (95% CI, 2.01–4.03; *p* < 0.01) between children who hesitated at all doses and those fully vaccinated. The cumulative meta-analysis revealed a decreasing temporal change in pertussis ORs (Fig. S1b, Additional File). Pertussis ORs were similar between North America and Eastern Asia (Table 2).

Stratified by vaccine hesitancy status, children with pertussis were 4.05 (95%CI, 3.15–5.20; ${I}^{2}=$ 0%) times more likely to be unvaccinated at all doses compared to those without pertussis. Subgroup analysis of under-vaccinated and vaccination-delayed children at all doses generated ORs of 2.95 (95% CI, 1.61–5.40; ${I}^{2}=$ 63%) and 1.18 (95% CI, 0.74–1.89; ${I}^{2}=$ 57%), respectively.

Stratified by the number of vaccine doses, children with pertussis were 3.79 (95% CI, 2.66–5.40; ${I}^{2}=$ 0%) and 7.79 (95% CI, 6.82–8.90; ${I}^{2}=$ 6%) times more likely to be unvaccinated with two and three doses than those without, respectively. The highest estimated OR was between children unvaccinated for four doses and children fully vaccinated (OR = 14.26; 95%CI, 7.62–26.70; ${I}^{2}=$ 87%). Children under-vaccinated with four doses also had the highest OR, at 8.21 (95% CI, 1.26–53.49; ${I}^{2}=$ 61%) among all under-vaccinated groups. Stratified by the vaccine dose number, the estimated OR of children under-vaccinated in the primary series was higher than that of children under-vaccinated at dose number 4 (Table 2). Conversely, the estimated OR of children under-vaccinated at dose numbers 5 and 4&5 was higher than the primary series (Table 2). Subgroup analysis generated an insignificant OR of 1.40 (95% CI, 0.63–3.16; ${I}^{2}=$ 59%) between children delayed in the primary series and children fully vaccinated. The lowest OR was between children delayed at dose number 4 with a value of 0.80 (95% CI, 0.48–1.34).

Sensitivity analyses showed that the pooled estimates were robust in unvaccinated children and those under-vaccinated with one and two doses. However, the OR of children under-vaccinated over 3 doses decreased (*p* > 0.05) when the study conducted by Glanz [44] was omitted (Table S3, Additional File).

### Maternal vaccine hesitancy and pertussis

Meta-analysis of the 10 included maternal vaccine hesitancy studies generated a random-effects pooled pertussis OR of 5.63 (95% CI, 3.87–8.18; *p* = 0.02) in infants. The cumulative meta-analysis showed a decreasing temporal change in pertussis ORs in infants (Fig. S1c, Additional File).

Stratified by study population age, the estimated OR was higher in infants $\le$ 2 months old(OR = 6.05; 95% CI, 4.31–8.50; ${I}^{2}=$ 12%) than in infants $\le$ 3 months (OR = 5.14; 95% CI, 1.95–13.52; ${I}^{2}=$ 80%). The significant heterogeneity in infants $\le$ 3 months old may be due to the quality of included studies. The maternal vaccine hesitant group received postpartum vaccinations in the study conducted by Winter et al. [53], which might introduce bias in the analysis. Sensitivity analysis showed that the pooled estimates were robust in infants $\le$ 2 months old, and a higher (*p* > 0.05) pooled OR was found in infants $\le$ 3 months old by omitting the study conducted by Winter et al. [53] (Table S3, Additional File).

### Pertussis vaccination effectiveness

Stratified by study outcomes in pregnant women, the estimated VE of maternal pertussis-containing vaccination was 89.83% (95% CI, 86.44%-93.35%; ${I}^{2}=$ 0%) and 80.60% (95%CI, 68.20%–95.26%; ${I}^{2}=$ 8%; Table 3) in preventing infants from pertussis infection and hospitalization, respectively. Stratified by the timing of maternal vaccine administration, the estimated VE of vaccination in the third trimester of pregnancy was slightly lower (*p* > 0.05) than that of vaccination administered at any point during pregnancy. Stratified by the number of vaccine doses, the estimated VE of childhood vaccination was the lowest at the first dose in preventing children from pertussis infection (VE = 66.25%; 95%CI, 51.43%–85.35%; ${I}^{2}=$ 48%). Sensitivity analyses showed that the estimated VEs were robust (Fig. S2, S3, Additional File).

**Table 3:** Vaccine effectiveness (VE) of vaccination among different subgroups

Vaccine Population	Variables	No. of studies	Estimated VE and 95% CI	${I}^{2}$
Pregnant Women	**Study Outcome**			
	Pertussis case	12 [7–9, 52–60]	89.83 (86.44, 93.35)	0%
	Hospitalization	2 [9, 55]	80.60 (68.20, 95.26)	8%
	**Timing of vaccine administration**			
	The third trimester	7 [8, 9, 52, 53, 57, 58, 60]	89.56 (85.66, 93.64)	0%
	Any point during pregnancy	7 [7, 8, 53, 54, 56, 58, 59]	90.64 (83.99, 93.35)	0%
Children	**Vaccine doses**			
	1	3 [45, 48, 50]	66.25 (51.43, 85.35)	48%
	2	3 [45, 48, 50]	80.12 (73.11, 87.80)	0%
	3	2 [45, 46, 48]	89.71 (86.87, 92.65)	74%
	4	1 [46]	95.00 (92.52, 97.55)	NA
	5	1 [45]	89.00 (82.62, 95.88)	NA
	All	4 [46, 47, 49, 50]	84.73 (78.41, 91.55)	0%
	**Study Outcome**			
	Pertussis case	6^45–50^	86.45 (83.45, 89.55)	71%
	Hospitalization	1^48^	92.04 (87.01, 97.36)	66%

## Discussion

We found a significant association between vaccine hesitancy and higher pertussis ORs in infants and children. At the population level, maternal and childhood vaccinations are highly effective at reducing the rate and severity of pertussis infection in infants and children.

Childhood vaccine hesitancy is an essential barrier to preventing pertussis in children, regardless of vaccine coverage. Although evidence indicated that the pertussis ORs were higher before 2018, recent studies still advocate that vaccine hesitancy is an important factor behind significantly higher pertussis risks in infants and children. Children with pertussis are more likely to be unvaccinated than under-vaccinated. Several factors may lead to childhood unvaccination, including nonmedical exemptions (such as philosophical, personal belief, or religious exemptions), cultural norms, unavailability of vaccination appointments, and hesitance toward vaccine providers [18]. Nonmedical exemptions were associated with significantly higher pertussis-related risks in children [14, 15, 28]. However, meta-analyses on this topic could not be conducted due to the limited number of population-level studies available. Further studies should be conducted to determine the relationship between pertussis and various factors related to childhood unvaccination/under-vaccination. The high heterogeneity in the subgroup analysis regarding the number of unvaccinated doses may be due to different vaccine schedules across different regions. Different recommended ages for children to take their first and fourth dose of vaccines against pertussis in immunization programs in the United States [45] and New Zealand [48] may explain the high heterogeneity in the estimated effect of 1 and 4 doses childhood under-vaccination.

The dose–effect relationship exists in childhood pertussis vaccine hesitancy. Pertussis ORs increased gradually as more vaccine doses were missing. Vaccine hesitancy over boosters may lead to more adverse effects in children. VE was the lowest at the first dose and gradually increased with the dose number, indicating a more significant preventive effect of pertussis-containing vaccines for older children. Waning immunity and vaccine hesitancy leading to the absence or late administration of boosters may result in more severe cases of pertussis in older children and higher VE of boosters [18]. However, for each dose, the effect of waning immunity may be insignificant because of the short follow-up periods of studies included in our analysis. The 4^th^ dose showed the highest VE, but the population-level data were too limited to conduct a meta-analysis. Delayed early vaccination seemed to be unimportant due to the relatively low VE and dose–effect relationship. However, deferring early vaccines may lead to missed or delayed vaccinations [61]. Together, we advocate that children should receive up-to-time pertussis-containing vaccinations – both primary series and subsequent boosters – to reduce regional and global pertussis outbreaks.

Maternal vaccine hesitancy was associated with significantly higher pertussis risks in infants. At the population level, maternal vaccination had significant protective effects both on infants too young to be vaccinated ($\le$ 2 months) and on infants eligible for their 1^st^ dose of pertussis-containing vaccination ($\le$ 3 months), with higher protective effects in the former group. This notion is also supported by the results of previous clinical trials [62–64]. Barug et al. reported a higher geometric mean concentration of pertussis toxin antibodies in 3-month-old infants whose mothers received maternal Tdap compared to that in those whose mothers declined [63]. Even when maternal vaccination failed to prevent infants from contracting pertussis, infants whose mothers received the maternal vaccine had a significantly lower risk of hospitalization. Thus, maternal vaccination is important for preventing infants $\le$ 3 months old from pertussis infections and reducing the severity of the disease if contracted. Maternal VE was high in infants, regardless of the vaccine administered timing. Previous studies reported no increased risks of adverse events among women who received maternal pertussis-containing vaccines and their infants [62–65]. Together, maximizing pertussis-containing vaccine uptake during pregnancy should be promoted worldwide, particularly in countries with re-emerging pertussis outbreaks.

We observed that vaccine delay was not significantly associated with higher pertussis risks in children. The high heterogeneity of meta-analysis may be explained by different population characteristics and government policies between Taiwan and the United States [23, 40]. A lower pertussis risk was indicated when the 4^th^ dose was delayed, but related studies were too limited to perform a full meta-analysis. Delaying childhood pertussis-containing vaccination may reduce the incidence of allergic diseases in infants and children [66, 67], indicating that delaying childhood vaccination may carry potential benefits. Because maternal vaccinations may reduce pertussis risks in infants $\le$ 3 months, infants whose mothers received maternal vaccinations may be able to delay the administration of their 1^st^ dose of pertussis-containing vaccination. However, further evidence is needed to verify this notion. More studies are needed to determine the specific and accurate associations between vaccine delays, including of different doses, and the risks of pertussis infection in children. We suggest that a clearer and standardized definition of vaccine delay and under-vaccination should be adopted for future studies on the topic, which may help with generating robust and comparable results.

Limitations also exist. First, different countries have different recommended vaccination ages for children, which may introduce high heterogeneity in our meta-analyses. Due to these differences, we were unable to evaluate the association between childhood vaccine hesitancy and pertussis risks in different age groups. Second, few studies investigated the specific effects of vaccine hesitancy during pertussis outbreaks; therefore, we could not assess differences in the effects of vaccine hesitancy on the risks of developing pertussis in children during the outbreak vs. non-outbreak years. Third, seven studies had NOS < 7, indicating potential defects in study design that may affect the accuracy of our meta-analysis. Lastly, the effects of psychological state or decision-making aspects of vaccine hesitancy on pertussis were not investigated because of limited studies in this study.

## Conclusion

We found an overall higher pertussis risk in infants and children who were unvaccinated or under-vaccinated and helped to fill in the knowledge gap in the association between pertussis vaccine delay and pertussis risks. The results provide a context for the promotion of maternal pertussis vaccination and indicate a possibility of childhood pertussis vaccination delay on the first dose. Improving maternal vaccine acceptance and up-to-date childhood vaccines are suggested to achieve better control over pertussis.

## Supplementary Information

**Additional file 1:** **Fig. S1.** Temporal changes in the odds ratios of pertussis under the effect of a) vaccine hesitancy, b) childhood vaccine hesitancy, and c) maternal vaccine hesitancy at all doses. **Fig. S2.** Sensitivity analysis of subgroup analyses pooled vaccine effectiveness (VE) estimates between the maternal fully vaccinated and vaccine hesitancy groups. **Fig. S3.** Sensitivity analysis of subgroup analyses pooled vaccine effectiveness (VE) estimates between the childhood fully vaccinated and vaccine hesitancy groups. **Table S1.** Characteristics of the studies included in the meta-analysis. **Table S2.** Quality evaluation results of NOS included in the study. **Table S3.** Sensitive analyses of pooled meta-analysis estimates.

## Data Availability

The data that support the findings of this study are from published studies. The data are publicly available and the extracted data are reported in the Supplement (Table S1, Additional File).

## Abbreviations

OR: Odds ratio
RR: Risk ratio
VE: Vaccine effectiveness
CI: Confidence interval
NOS: Newcastle–Ottawa Scale
PICOS: Population, Intervention, Control, Outcome, Study design
WHO: World Health Organization
