# Supplementary material for: The association between vaccine hesitancy and pertussis: a systematic review and meta-analysis
Source: Ital J Pediatr. 2023 Jul 13;49:81. doi: 10.1186/s13052-023-01495-8 (PMC10339594; doi:10.1186/s13052-023-01495-8)
Supplement: Supplementary file 1 — Additional file 1: Fig. S1. Temporal changes in the odds ratios of pertussis under the effect of a) vaccine hesitancy, b) childhood vaccine hesitancy, and c) maternal vaccine hesitancy at all doses. Fig. S2. Sensitivity analysis of subgroup analyses pooled vaccine effectiveness (VE) estimates between the maternal fully vaccinated and vaccine hesitancy groups. Fig. S3. Sensitivity analysis of subgroup analyses pooled vaccine effectiveness (VE) estimates between the childhood fully vaccinated and vaccine hesitancy groups. Table S1. Characteristics of the studies included in the meta-analysis. Table S2. Quality evaluation results of NOS included in the study. Table S3. Sensitive analyses of pooled meta-analysis estimates. [file 13052_2023_1495_MOESM1_ESM.docx]

**The Association between Vaccine Hesitancy and Pertussis: A Systematic Review and Meta-analysis**

Yuning Wang^1,2^, Naiyang Shi^1,2^, Qiang Wang^1,2^, Liuqing Yang^1,2^, Tingting Cui^1,2^, Hui Jin^1,2^

**Table of Contents**

**Fig. S1** Temporal changes in the odds ratios of pertussis under the effect of a) vaccine hesitancy, b) childhood vaccine hesitancy, and c) maternal vaccine hesitancy at all doses.

**Fig. S2** Sensitivity analysis of subgroup analyses pooled vaccine effectiveness (VE) estimates between the maternal fully vaccinated and vaccine hesitancy groups.

**Fig. S3** Sensitivity analysis of subgroup analyses pooled vaccine effectiveness (VE) estimates between the childhood fully vaccinated and vaccine hesitancy groups.

**Table S1** Characteristics of the studies included in the meta-analysis

**Table S2**. Quality evaluation results of NOS included in the study

**Table S3**. Sensitive analyses of pooled meta-analysis estimates


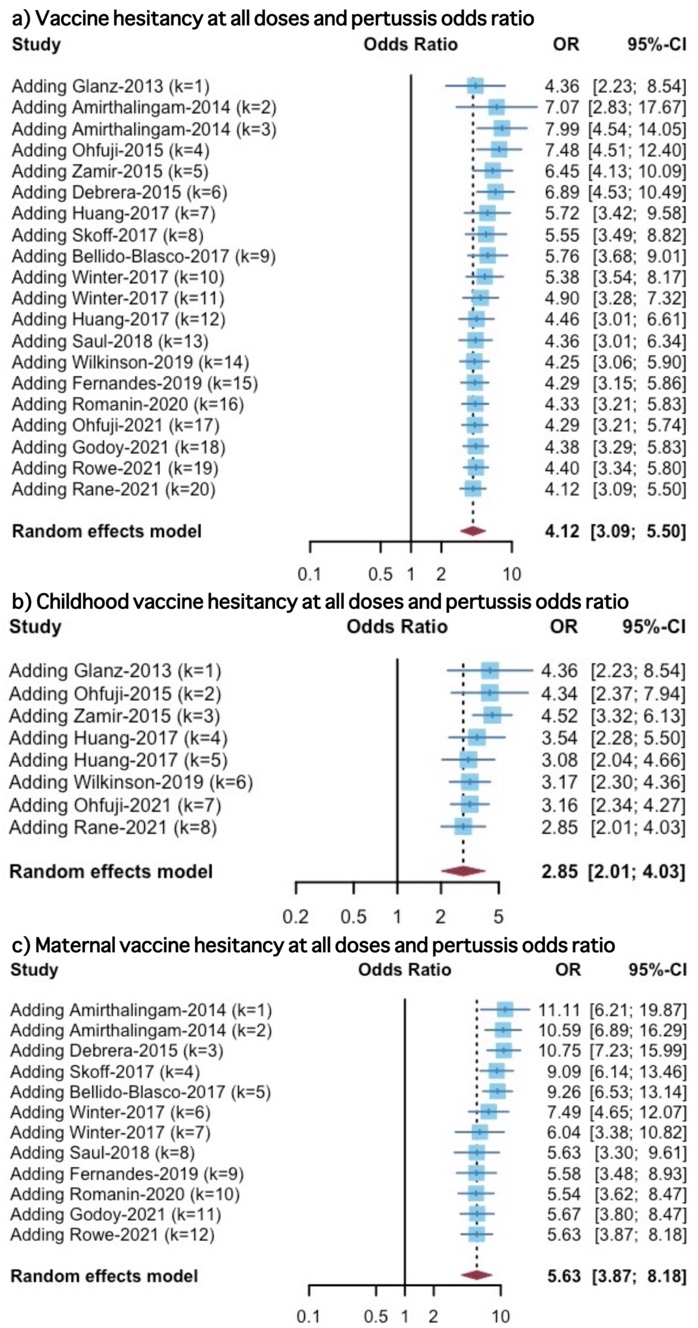


**Fig. S1** Temporal changes in the odds ratios of pertussis under the effect of a) vaccine hesitancy, b) childhood vaccine hesitancy, and c) maternal vaccine hesitancy at all doses.


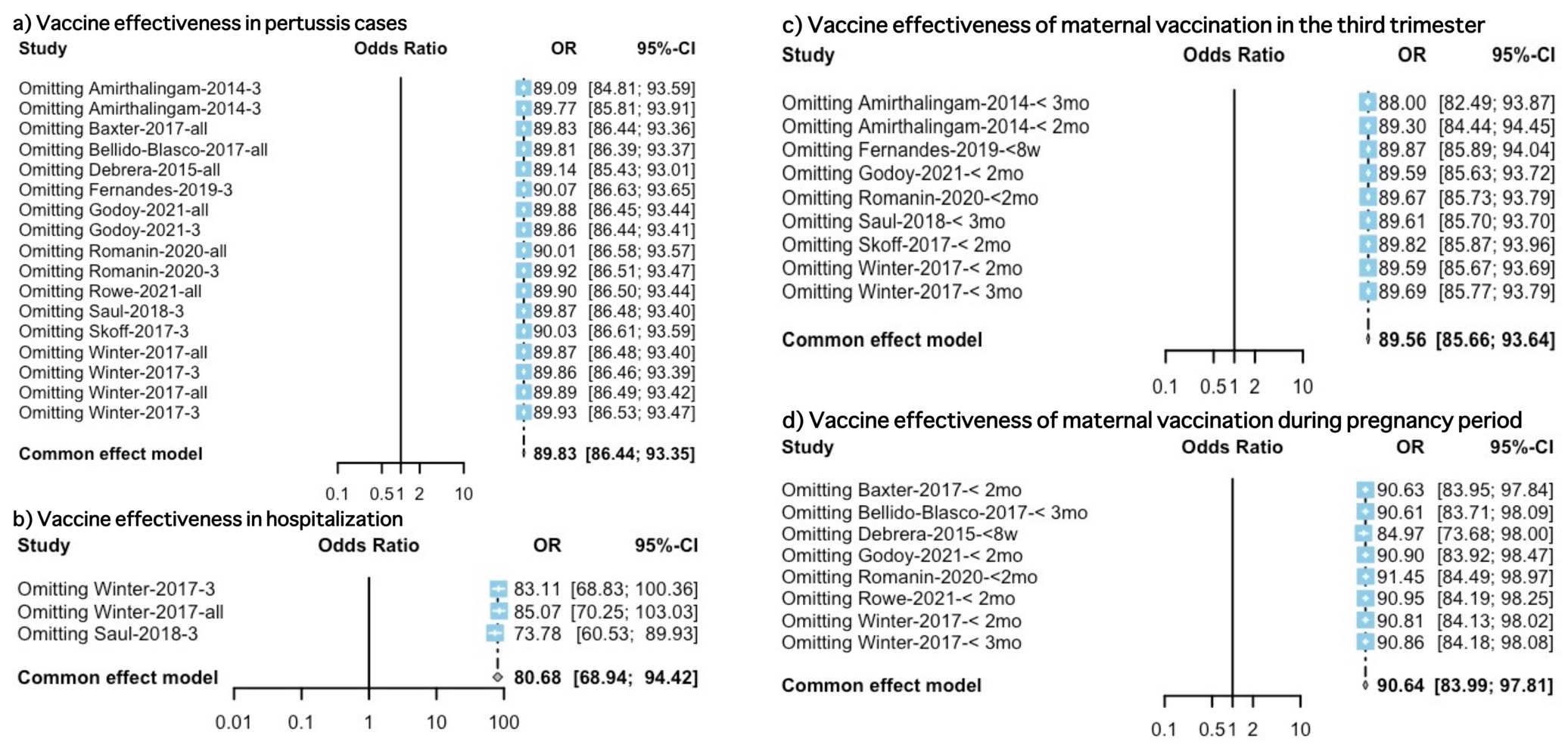


**Fig. S2** Sensitivity analysis of subgroup analyses pooled vaccine effectiveness (VE) estimates between the maternal fully vaccinated and vaccine hesitancy groups.


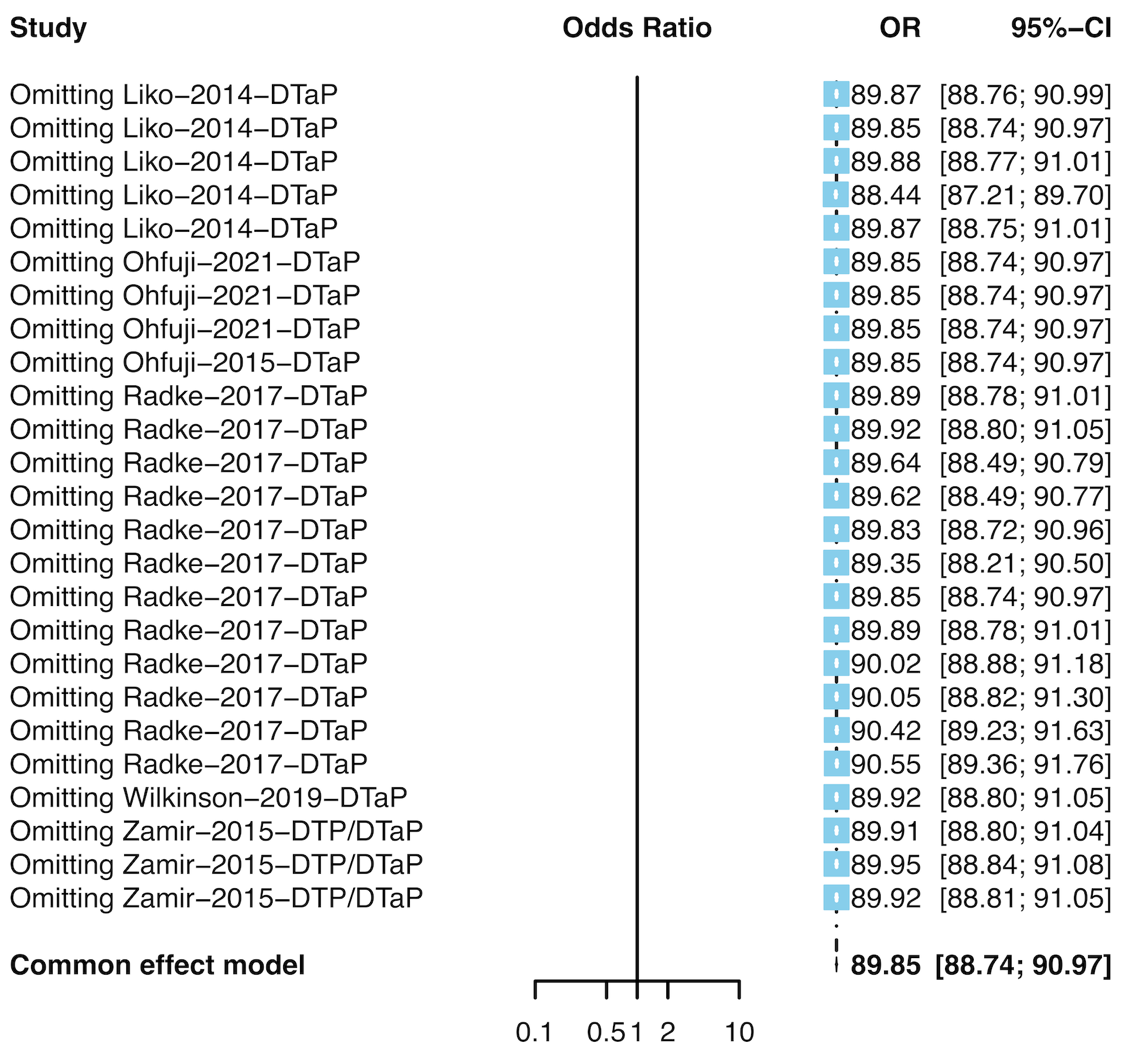


**Fig. S3** Sensitivity analysis of subgroup analyses pooled vaccine effectiveness (VE) estimates between the childhood fully vaccinated and vaccine hesitancy groups.

**Table S1** Characteristics of the studies included in the meta-analysis

| Study, First Authors, and year | Location | Region | Design | Vaccine population | Study population | Sample size | Vaccine hesitancy status | OR (95% CI) | Vaccine effectiveness % (95% CI) | Outcome | NOS |
| --- | --- | --- | --- | --- | --- | --- | --- | --- | --- | --- | --- |
| **Childhood Vaccination** |  |  |  |  |  |  |  |  |  |  |  |
| Glanz (2013) | Multisites in US | North America | Matched case-control study | Children | Children <= 36 months | 360 | Undervaccinated | 1 dose: 2.25 (0.97-5.24); 2 doses: 3.41 (0.89-13.05); 3 doses: 18.56 (4.92-69.95); 4 doses: 28.38 (3.19-252.63); All doses: 4.36 (2.23-8.55) | NA | Pertussis cases | 8 |
| Bailon (2016) | Peru | South America | Cross-sectional study | Children | Children < 18 months | 849 | Undervaccinated | Dose 4&5: 4.34 (2.27-9.09) | NA | Pertussis cases | 6 |
| Huang (2016) | Taiwan | Eastern Asia | Matched case-control study | Children | Children <= 36 months | 3045 | Undervaccinated; Vaccine delayed | Undervaccinated:  1 dose: 1.84 (1.17-2.89); 2 doses: 2.80 (1.44-5.44); 3 doses: 3.85 (1.93-7.67); 4 doses: 3.79 (1.19-12.07); all doses: 2.28 (1.57-3.31);  Dose 1&2&3: 2.25 (1.71-3.71); Dose 4: 1.02 (0.33-3.15).  Vaccine delayed:  all doses: 1.93 (1.21-3.06). | NA | Pertussis cases | 8 |
| Liko (2014) | US | North America | Retrospective cohort study | Children | Children <= 47 months | 182719 | Undervaccinated; Unvaccinated | Undervaccinated: 1 dose: 2.00 (0.60-6.80); 2 doses: 1.80 (0.80-3.90); 3 doses: 3.00 (1.90-4.80). Unvaccinated: 1 dose: 3.70 (1.80-7.80); 2 doses: 3.50 (1.10-11.70); 3 doses: 7.10 (3.60-14.10); 4 doses: 20.60 (13.30-31.80). | Unvaccinated: 1 dose: 73.0 (43.0-87.0); 2 doses: 72.0 (6.0-91.0); 3 doses: 86.0 (72.0-93.0); 4 doses: 95.0 (92.0-97.0). | Pertussis cases | 6 |
| Ohfuji (2020) | Multisites in Japan | Eastern Asia | Case-control | Children | Children <= 23 months | 145 | Unvaccinated | all doses: 3.13 (0.56-16.70) | 3 doses: 84 (-105-99);  4 doses: 90 (-20-99);  All doses: 88 (-33-99). | Pertussis cases | 8 |
| Ohfuji (2015) | Multisites in Japan | Eastern Asia | Case-control | Children | Children <= 23 months | 145 | Unvaccinated | all doses: 4.23 (1.05-17.11) | All doses: 80 (3-69). | Pertussis cases | 6 |
| Radke (2016) | New Zealand | Australia | Case-control | Children | Children < 7 years old | 55444 | Unvaccinated | Unvaccinated: 1 dose: 1.28 (1.05-1.56); 2 doses: 3.82 (2.63-5.53); 3 doses: 8.33 (6.41-10.81), 9.14 (7.32-11.41), 7.43 (5.91-9.33), 6.71 (5.39-8.35); 4 doses: 10.55 (8.83-12.61). | Hospitalization: 1 dose: 43.0 (21.0-58.0); 2 doses: 84.0 (72.0-91.0); 3 doses: 93.0 (87.0-97.0), 94.0 (87.0-97.0), 92.0 (79.0-97.0). Non-hospitalization: 1 dose: 28.0 (-23.0-58.0); 2 doses: 70.0 (47.0-83.0); 3 doses: 87.0 (82.0-91.0), 89.0 (86.0-91.0), 86.0 (83.0-89.0); | Pertussis cases | 7 |
| Rane (2021) | Washington, US | North America | Retrospective cohort study | Children | Children < 9 years old | 316404 | Undervaccinated; Vaccine delayed | Undervaccinated:  Dose 1&2&3 dose: 4.80 (3.10-7.60); Dose 4: 3.20 (2.30-4.50); Dose 5: 4.60 (2.60-8.20); 1 dose: 3.50 (2.30-5.50). Vaccine delayed:  all doses: 0.80 (0.30-2.20); Dose 4: 0.80 (0.50-1.40); Dose 5: 1.30 (0.50-3.60). | NA | Pertussis cases | 9 |
| Wilkinson (2019) | Canada | North America | Matched case-control study | Children | Children < 9 years old | 2730 | Unvaccinated | all doses: 3.57 (2.50-5.26) | All doses: 72 (60-81) | Pertussis cases | 7 |
| Zamir (2015) | Isreal | Western Asia | Matched case-control study | Children | Children < 12 months | 1268 | Unvaccinated | all doses: 4.58 (3.21-6.53) | 1 dose: 72.9 (58.9-82.2); 2 doses: 76.1 (60.6-85.6); 3 doses: 84.4 (72.2-91.3). | Pertussis cases | 8 |
| **Maternal Vaccination** |  |  |  |  |  |  |  |  |  |  |  |
| Dabrera (2014) | UK | Europe | Case-control | Pregnant women | Infants $\leq$ 2 months | 113 | Unvaccinated | 11.7 (4.35-33.3) | all time: 93.0 (81.0-97.0) | Pertussis cases | 7 |
| Fernandes (2019) | Brazil | South America | Unmatched case-control study | Pregnant women | Infants $\leq$ 2 months | 290 | Unvaccinated | 5.18 (2.27-11.9) | 3rd trimester: 80.7 (55.9-91.6) | Pertussis cases | 7 |
| Godoy (2021) | Multisites in Spain | Europe | Matched case-control study | Pregnant women | Infants $\leq$2 months | 171 | Unvaccinated | 8.33 (2.38-33.3) | 3rd trimester: 88.5 (54.9-97.1); all time: 88.0 (58.3-96.5) | Pertussis cases | 8 |
| Romanin (2020) | Multisites in Argetina | South America | Matched case-control study | Pregnant women | Infants $\leq$ 2 months | 371 | Unvaccinated | 5.18 (2.09-12.82) | 3rd trimester: 75.4 (49.8-88); all time: 80.7 (46.4-94.4) | Pertussis cases | 7 |
| Skoff (2017) | US | North America | Case-control | Pregnant women | Infants $\leq$ 2 months | 775 | Unvaccinated | 4.48 (1.93-10.42) | 3rd trimester: 77.7 (48.3-90.4) | Pertussis cases | 6 |
| Bellido-Blasco (2017) | Spain | Europe | Matched case-control study | Pregnant women | Infants $\leq$ 3 months | 88 | Unvaccinated | 11.00 (2.30-52.63) | all time: 90.9 (56.6-98.1) | Pertussis cases | 7 |
| Rowe (2021) | Australia | Australia | Retrospective cohort study | Pregnant women | Infants $\leq$ 2 months | 187962 | Unvaccinated | 5.02 (1.59-15.87) | all time: 80.1 (37.1-93.7) | Pertussis cases | 7 |
| Saul (2018) | Australia | Australia | Matched case-control study | Pregnant women | Infants $\leq$ 3 months | 234 | Unvaccinated | 3.23 (1.15-9.09) | Pertussis: 69.0 (13.0-89.0); Hospitalization: 94.0 (59.0-99.0) | Pertussis cases & hospitalization | 7 |
| Winter (2017) | US | North America | Retrospective cohort study | Pregnant women | Infants $\leq$ 2 months; Infants $\leq$ 3 months | 74504 | Unvaccinated | Infants $\leq$ 2 months: 2.78 (1.12-6.67); Infants $\leq$ 3 months: 2.13 (1.1-4.17) | 3rd trimester:  $\leq$ 2 months: 85.4 (33.0-96.7); $\leq$ 3 months: 71.6 (29.6-88.6) all time:  $\leq$ 2 months: 63.8 (10.6-85.4); $\leq$ 3 months: 53.0 (8.2-75.9) | Pertussis cases | 6 |
| Winter (2017) | US | North America | Retrospective cohort study | Pregnant women | Infants $\leq$ 2 months | 420 | Unvaccinated | NA | 3rd trimester: 75.4 (49.8-88); all time: 72.3 (49.0-85.0) | Hospitalization due to pertussis | 6 |
| Amirthalingam (2014) | UK | Europe | Retrospective cohort study | Pregnant women | Infants $\leq$ 2 months; Infants $\leq$ 3 months | 90 | Unvaccinated | Infants $\leq$ 2 months: 10.00 (5.56-20.00); Infants $\leq$ 3 months: 11.11 (6.25-20.00) | 3rd trimester: $\leq$ 2 months: 90.0 (82.0-95.0); $\leq$ 3 months: 91.0 (84.0-95.0) | Pertussis cases | 6 |
| Baxter (2017) | US | North America | Retrospective cohort study | Pregnant women | Infants $\leq$ 2 months | 148981 | Unvaccinated | NA | all time: 91.94 (19.5-99.1) | Pertussis cases | 7 |

NA = not applicable

**Table S2**. Quality evaluation results of NOS included in the study

1. Case-control studies

| Study | Selection | | | | Comparability | Exposure | | | Quality score |
| --- | --- | --- | --- | --- | --- | --- | --- | --- | --- |
|  | Adequate definition of cases | Representativeness of the cases | Selection of controls | Definition of controls | Important factors controlled for the comparability between the controls and cases | Ascertainment of exposure | Same method of ascertainment for cases | Non-response rate |  |
| Glanz (2013) | 1 | 1 | 1 | 1 | 2 | 1 | 1 | 0 | 8 |
| Huang (2016) | 1 | 1 | 1 | 1 | 2 | 1 | 1 | 0 | 8 |
| Ohfuji (2020) | 1 | 1 | 1 | 1 | 2 | 1 | 1 | 0 | 8 |
| Ohfuji (2015) | 1 | 1 | 0 | 1 | 2 | 0 | 1 | 0 | 6 |
| Wilkinson (2019) | 1 | 1 | 1 | 1 | 2 | 0 | 1 | 0 | 7 |
| Zamir (2015) | 1 | 1 | 1 | 1 | 2 | 1 | 1 | 0 | 8 |
| Dabrera (2014) | 1 | 1 | 1 | 1 | 2 | 0 | 1 | 0 | 7 |
| Fernandes (2019) | 1 | 1 | 1 | 1 | 1 | 1 | 1 | 0 | 7 |
| Godoy (2021) | 1 | 1 | 1 | 1 | 2 | 1 | 1 | 0 | 8 |
| Romanin (2020) | 1 | 1 | 1 | 1 | 2 | 0 | 1 | 0 | 7 |
| Skoff (2017) | 1 | 1 | 1 | 1 | 1 | 0 | 1 | 0 | 6 |
| Bellido-Blasco (2017) | 1 | 1 | 1 | 1 | 1 | 1 | 1 | 0 | 7 |
| Saul (2018) | 1 | 1 | 1 | 1 | 2 | 0 | 1 | 0 | 7 |
| Radke (2016) | 0 | 1 | 1 | 1 | 2 | 1 | 1 | 0 | 7 |

1. Cohort studies

| Study | Selection | | | | Comparability | Outcome | | | Quality score |
| --- | --- | --- | --- | --- | --- | --- | --- | --- | --- |
|  | Representativeness of the exposed cohort | Selection of the non-exposed cohort | Ascertainment of exposure | Demonstration that outcome of interest was not present at start of study | Comparability of cohorts on the basis of the design or analysis | Assessment of outcome | Was follow-up long enough for outcomes to occur | Adequacy of follow-up of cohorts |  |
| Bailon (2016) | 1 | 1 | 1 | 1 | 1 | 1 | 0 | 0 | 6 |
| Liko (2014) | 1 | 1 | 1 | 1 | 1 | 1 | 0 | 0 | 6 |
| Rane (2021) | 1 | 1 | 1 | 1 | 2 | 1 | 1 | 1 | 9 |
| Rowe (2021) | 1 | 1 | 1 | 1 | 2 | 1 | 0 | 0 | 7 |
| Winter (2017) | 1 | 1 | 1 | 0 | 2 | 1 | 0 | 0 | 6 |
| Winter (2017) | 1 | 1 | 1 | 0 | 2 | 1 | 0 | 0 | 6 |
| Amirthalingam (2014) | 1 | 1 | 1 | 1 | 1 | 1 | 0 | 0 | 6 |
| Baxter (2017) | 1 | 1 | 1 | 0 | 2 | 1 | 0 | 1 | 7 |

**Table S3**. Sensitive analyses of pooled meta-analysis estimates

| Vaccine population | Vaccine hesitancy Status | Variable | Study | OR | 95% CI | $I^{2}$ |
| --- | --- | --- | --- | --- | --- | --- |
| All | Vaccine hesitant | **Vaccine doses** |  |  |  |  |
|  |  | All | Omitting Glanz-2013 | 4.11 | [3.03; 5.58] | 70.3% |
|  |  |  | Omitting Huang-2017 | 4.32 | [3.21; 5.82] | 65.6% |
|  |  |  | Omitting Ohfuji-2021 | 4.14 | [3.09; 5.58] | 70.3% |
|  |  |  | Omitting Ohfuji-2015 | 4.12 | [3.06; 5.54] | 70.3% |
|  |  |  | Omitting Wilkinson-2019 | 4.18 | [3.04; 5.76] | 70.3% |
|  |  |  | Omitting Zamir-2015 | 4.10 | [2.98; 5.64] | 69.7% |
|  |  |  | Omitting Debrera-2015 | 3.93 | [2.94; 5.25] | 67.8% |
|  |  |  | Omitting Fernandes-2019 | 4.07 | [3.01; 5.50] | 70.1% |
|  |  |  | Omitting Godoy-2021 | 4.03 | [3.00; 5.41] | 69.7% |
|  |  |  | Omitting Romanin-2020 | 4.08 | [3.02; 5.50] | 70.1% |
|  |  |  | Omitting Skoff-2017 | 4.11 | [3.04; 5.55] | 70.3% |
|  |  |  | Omitting Bellido-Blasco-2017 | 4.02 | [3.00; 5.38] | 69.4% |
|  |  |  | Omitting Rowe-2017 | 4.09 | [3.04; 5.51] | 70.2% |
|  |  |  | Omitting Saul-2018 | 4.17 | [3.09; 5.62] | 70.3% |
|  |  |  | Omitting Winter-2017 | 4.21 | [3.11; 5.68] | 70.1% |
|  |  |  | Omitting Winter-2017 | 4.29 | [3.19; 5.79] | 68.8% |
|  |  |  | Omitting Amirthalingam-2014 | 3.81 | [2.91; 5.01] | 61.4% |
|  |  |  | Omitting Amirthalingam-2014 | 3.87 | [2.92; 5.14] | 65.0% |
|  |  |  | Omitting Huang-2017 | 4.36 | [3.26; 5.83] | 65.2% |
|  |  |  | Omitting Glanz-2013 | 4.40 | [3.34; 5.80] | 64.8% |
|  |  |  | **Pooled Estimate** | **4.12** | **[3.09; 5.50]** | **68.7%** |
| Children | Unvaccinated | **Vaccine doses** |  |  |  |  |
|  |  | All | Omitting Ohfuji-2021 | 4.07 | [3.12; 5.31] | 0.0% |
|  |  |  | Omitting Ohfuji-2015 | 4.03 | [3.05; 5.34] | 0.0% |
|  |  |  | Omitting Wilkinson-2019 | 4.49 | [3.17; 6.35] | 0.0% |
|  |  |  | Omitting Zamir-2015 | 3.59 | [2.52; 5.11] | 0.0% |
|  |  |  | **Pooled Estimate** | **4.04** | **[3.10; 5.27]** | **0.0%** |
|  | Under-vaccinated | **Vaccine doses** |  |  |  |  |
|  |  | 1 | Omitting Glanz-2013 | 2.48 | [1.58; 3.89] | 52.1% |
|  |  |  | Omitting Huang-2017 | 2.95 | [1.91; 4.56] | 0.0% |
|  |  |  | Omitting Liko-2014 | 2.49 | [1.64; 3.80] | 51.3% |
|  |  |  | Omitting Rane-2021 | 1.93 | [1.32; 2.83] | 0.0% |
|  |  |  | **Pooled estimate** | **2.45** | **[1.70; 3.54]** | **29.3%** |
|  |  | 2 | Omitting Glanz-2013 | 2.32 | [1.33; 4.04] | 0.0% |
|  |  |  | Omitting Huang-2017 | 2.17 | [1.01; 4.64] | 0.0% |
|  |  |  | Omitting Liko-2014 | 2.91 | [1.60; 5.29] | 0.0% |
|  |  |  | **Pooled estimate** | **2.45** | **[1.46; 4.10]** | **0.0%** |
|  |  | 3 | Omitting Glanz-2013 | 3.25 | [2.18; 4.84] | 0.0% |
|  |  |  | Omitting Huang-2017 | 6.62 | [1.21; 36.09] | 84.5% |
|  |  |  | Omitting Liko-2014 | 7.53 | [1.75; 32.36] | 76.5% |
|  |  |  | **Pooled estimate** | **5.17** | **[1.84; 14.53]** | **69.1%** |
| Pregnant women | Unvaccinated | **Study population age** |  |  |  |  |
|  |  | Infants $\leq$2 months | Omitting Debrera-2015 | 5.67 | [4.04; 7.96] | 3.6% |
|  |  |  | Omitting Fernandes-2019 | 6.16 | [4.14; 9.16] | 22.9% |
|  |  |  | Omitting Godoy-2021 | 5.88 | [4.05; 8.54] | 22.4% |
|  |  |  | Omitting Romanin-2020 | 6.14 | [4.15; 9.08] | 23.2% |
|  |  |  | Omitting Skoff-2017 | 6.32 | [4.31; 9.27] | 18.4% |
|  |  |  | Omitting Rowe-2021 | 6.11 | [4.18; 8.95] | 23.5% |
|  |  |  | Omitting Winter-2017 | 6.83 | [4.88; 9.56] | 0.0% |
|  |  |  | Omitting Amirthalingam-2014 | 5.21 | [3.63; 7.49] | 0.0% |
|  |  |  | **Pooled estimate** | **6.05** | **[4.31; 8.50]** | **12.0%** |
|  |  | Infants $\leq$ 3 months | Omitting Bellido-Blasco-2017 | 4.34 | [1.40; 13.48] | 85.9% |
|  |  |  | Omitting Saul-2018 | 6.02 | [1.70; 21.29] | 85.8% |
|  |  |  | Omitting Winter-2017 | 7.46 | [3.22; 17.27] | 53.3% |
|  |  |  | Omitting Amirthalingam-2014 | 3.34 | [1.51; 7.40] | 45.2% |
|  |  |  | **Pooled estimate** | **5.14** | **[1.95; 13.52]** | **80.2%** |
